# Next-generation antiviral peptides: AI-driven design, translational delivery platforms, and future therapeutic directions

**DOI:** 10.1016/j.virusres.2025.199642

**Published:** 2025-10-15

**Authors:** Maryam Mashhadi Abolghasem Shirazi, Setareh Haghighat, Zahra Nikbakht, Elaheh Salimkia, Armity Kiumarsy

**Affiliations:** Department of Microbiology, TeMS.C., Islamic Azad University, Tehran, Iran

**Keywords:** Antiviral peptides, Artificial intelligence, Drug resistance, Machine learning, Generative adversarial networks, Large language models

## Abstract

Antiviral peptides (AVPs) are emerging as next-generation therapeutics due to their broad-spectrum activity, low toxicity, and ability to overcome drug resistance. The objective of this review is to provide an integrated perspective on AVP research, with particular emphasis on artificial intelligence (AI)-driven discovery, novel delivery strategies, and translational applications. We first summarize the origins, mechanisms, and structural diversity of AVPs. We then highlight recent advances in computational pipelines, including machine learning, deep learning, generative adversarial networks (GANs), large language models (LLMs), and reinforcement learning frameworks for *de novo* peptide design. Translational aspects are addressed by discussing novel delivery systems such as nanoparticles, hydrogels, and intranasal/inhalable formulations, as well as clinical trial examples (like, enfuvirtide (T-20), sifuvirtide, lactoferrin-based formulations, PAC-113). Finally, we explore future directions, including CRISPR- and mRNA-based peptide delivery and synergies with immune checkpoint inhibitors. By combining classical mechanisms with AI-driven design and innovative delivery platforms, this review underscores the potential of AVPs as versatile antiviral agents ready for clinical translation.

## Highlights

• Artificial intelligence and machine learning approaches for predictive AVP design.
• Novel translational strategies including nanoparticle-based, hydrogel, and intranasal delivery systems.
• Synthetic biology perspectives such as CRISPR-based expression systems and mRNA-encoded AVPs.
• Future clinical directions integrating AVPs with immune checkpoint inhibitors and current antivirals for synergistic effects.

## 1 Introduction

Viral infections remain one of the leading global health challenges, as demonstrated by recurrent outbreaks of influenza, *Human immunodeficiency virus* (HIV), herpesviruses, and more recently, the COVID-19 pandemic. Although vaccines and small-molecule antivirals have significantly reduced disease burden, their effectiveness is often limited by viral evolution, drug resistance, and safety concerns. This has stimulated interest in AVPs as a novel therapeutic class with unique features including broad-spectrum activity, low toxicity, and reduced likelihood of resistance development (Agarwal and Gabrani, 2021; Wang et al., 2016).

AVPs are naturally occurring or synthetic short amino acid sequences that exert antiviral effects through multiple mechanisms such as disrupting viral envelopes, inhibiting receptor binding, blocking genome replication, and modulating host immune responses (Jenssen et al., 2006; Zannella et al., 2022). Their versatility allows them to target both enveloped and non-enveloped viruses, making them attractive candidates for countering emerging viral threats. Several AVPs, including T-20 for HIV and PAC-113 for oral candidiasis, have advanced into clinical evaluation, providing proof-of-concept for their translational potential (Kilby et al., 1998; N. Falah et al., 2012).

In parallel, the rapid development of AI has transformed drug discovery, offering powerful methods to accelerate AVP research. Machine learning and deep learning approaches have been applied to predict peptide virus interactions, while recent generative models including (GANs), (LLMs), and reinforcement learning frameworks enable *de novo* peptide design with optimized stability, activity, and immunogenicity (Rao et al., 2019; Elnaggar et al., 2021; Das et al., 2021; Szymczak et al., 2025). Furthermore, explainable AI (XAI) techniques enhance interpretability, providing biological insights and improving translational reliability.

Given these advances, this review aims to provide an integrated overview of AVPs with a focus on three major aspects: (i) their structural diversity and antiviral mechanisms, (ii) the role of AI-driven platforms in their discovery and optimization, and (iii) novel delivery systems and translational strategies that can overcome barriers to clinical application. By combining classical antiviral research with computational and nanotechnological innovations, AVPs can be positioned as a next-generation therapeutic option to address both current and future viral challenges.

## 2 Overview of AVPs

Numerous studies have revealed that peptides from microbes have the capacity to fight viral infections, including HIV, *Herpes simplex virus* (HSV), and *Dengue Virus* (Urmi et al., 2023). AVPs can be found in nature within animals, plants, and microorganisms, while others are created artificially (Kuroki et al., 2021). They aim to interfere with various phases of the viral lifecycle, functioning effectively against viral infections (Vilas Boas et al., 2019). AVPs are recognized as promising treatments due to their rapid action in curbing viral spread (Kuroki et al., 2021; Vilas Boas et al., 2019). Their low toxicity means they pose minimal adverse effects on healthy cells and can be paired with antiviral medications to bolster treatment effectiveness (Meseko et al., 2023). Furthermore, AVPs have potential in preventive strategies, such as peptide-based vaccines (Urmi et al., 2023). The ongoing discovery of new natural AVPs, advancements in peptide design and production, the creation of stable antiviral compounds, and improved pharmacokinetic features contribute to the growing use of AVPs in treating viral diseases (Kuroki et al., 2021; Qureshi et al., 2015).

### 2.1 Sources of AVPs

AMPs are an integral part of the innate immune systems of many organisms, helping them fight off pathogens. In various animals, examples of these include defensins, cathelicidins, and histatins (Brice and Diamond, 2020). Venomous creatures, like scorpions, snakes, and spiders, also produce AMPs in their venom (Mata et al., 2017). Additionally, peptides from marine life such as mollusks, sponges, and algae are notable sources of bioactive peptides with various therapeutic potentials, making them particularly interesting for developing treatments against viral infections (Wang et al., 2020; Chen et al., 2014).

Plants generate various antiviral AMPs, which consist of 4 to 12 cysteine residues, like thionins, defensins, cyclotides, and lipid transfer proteins; these play a crucial role in defending against pathogens (Lima et al., 2022; Feyzyab et al., 2023). Found in all parts of the plant—roots, stems, leaves, flowers, and seeds—these peptides help protect against environmental threats (Nawrot et al., 2014). Moreover, plants can create peptides with antibacterial, antifungal, and antiparasitic properties. For instance, Pteleopsis suberosa has been shown to produce peptides targeting Helicobacter pylori, while Santolina chamaecyparissus yields substances effective against Candida spp., and cowpeas defend against Leishmania amazonensis (Tang et al., 2018).

Similarly, other microorganisms, including archaea, bacteria, and fungi, also develop peptides that exhibit antiviral properties. These peptides can either eliminate viruses directly or aid the host's immune defense against them, such as bacteriocins, lantibiotics, and fungal defensins (Zakaryan et al., 2021).

Insects and marine invertebrates produce AMPs like cecropin, drusinin, and tachyplesin, which possess antibacterial, antiviral, and antifungal capabilities found in epithelial cells, phagocytes, hemolymph, and hemocytes (Hajigha et al., 2024).

Vertebrates, including fish, birds, reptiles, and mammals, also create AMPs through the epithelial cells located in their skin, mucous membranes, and white blood cells (Edilia Avila, 2017). The roles of AMPs in these animals include fighting off microbial infections, managing adaptive immune responses, and supporting male reproductive health (Christophers and Schröder, 2022).

Among the AMPs identified in mammals are cathelicidins, defensins, and peptides produced by platelets and the liver, such as thrombocidin TC-1 (from platelets) and Hepcidin 20 (produced in the liver) (Ageitos et al., 2017). Inactive forms of cathelicidins are kept in leukocytes; when pathogens invade, these peptides get activated, processed, and released to exert their antibacterial effects (Tomasinsig and Zanetti, 2005). Notably, LL-37 has been highlighted among human AMPs; it has an alpha-helical structure and functions in immune regulation and antibacterial activity (Fabisiak et al., 2016). Defensins can be classified into three groups based on how their disulfide bonds are arranged: alpha, beta, and delta (Suarez-Carmona et al., 2015). In humans, both alpha and beta types are present. Alpha defensins are found in intestinal Paneth cells, neutrophils, and macrophages, while beta defensins are produced by epithelial cells, muscle fibers, cardiac cells, and leukocytes (Yang et al., 2022). The stability of defensins is reliant on these disulfide bonds, which enhance peptide structure and guard against proteases (Wanniarachchi et al., 2011).

By exploring the roles of natural AMPs and creating synthetic versions, it becomes possible to design and produce peptides with tailored structures and physicochemical properties aimed at fighting viral infections.

## 3 Mechanisms of antiviral activity

The antiviral mechanisms of AVPs are diverse and depend partly on whether the target virus is enveloped or non-enveloped. AVPs exert their effects through multiple mechanisms that can be broadly grouped into five major categories:

• Disruption of viral membranes or capsids: Direct interaction with viral envelopes or protein shells, leading to loss of integrity.
• Inhibition of receptor binding and viral entry: Blocking viral attachment to host cell receptors or preventing fusion.
• Interference with genome replication and protein synthesis: Inhibiting viral polymerases, proteases, or other enzymes essential for replication (Cole et al., 2002; Rodziewicz-Motowidło et al., 2010).
• Inhibition of viral assembly and release: Preventing capsid formation or budding from host cells.
• Immunomodulation: Enhancing host defense by stimulating cytokine production or activating innate immune cells (Tripathi et al., 2014; Van Harten et al., 2018).

While these mechanisms apply generally to many viruses, the relevance of each strongly depends on whether the virus is enveloped or non-enveloped:

• Enveloped viruses (like, HIV, influenza, HSV, SARS-CoV-2) are particularly susceptible to membrane-targeting AVPs that disrupt lipid bilayers or block envelope glycoproteins involved in entry (like, gp41, hemagglutinin (HA)). Fusion inhibitors and peptides interfering with viral polymerases are also effective in this category. For example, LL-37 and melittin destabilize viral envelopes, while EK1 and its derivatives block the HR1–HR2 interaction of coronavirus spike proteins, preventing fusion (Zannella et al., 2022; Lee et al., 2016; Xia et al., 2019; Xia et al., 2020).
• Non-enveloped viruses (like, *Adenovirus, Papillomavirus* (*HPVs*), *Enteroviruses*) lack lipid membranes and are instead targeted by AVPs that bind to or destabilize their capsid proteins, prevent receptor-mediated entry, or inhibit viral proteases required for genome replication and protein synthesis (Buck et al., 2013; Liu et al., 2022).

**Fig. 1:**
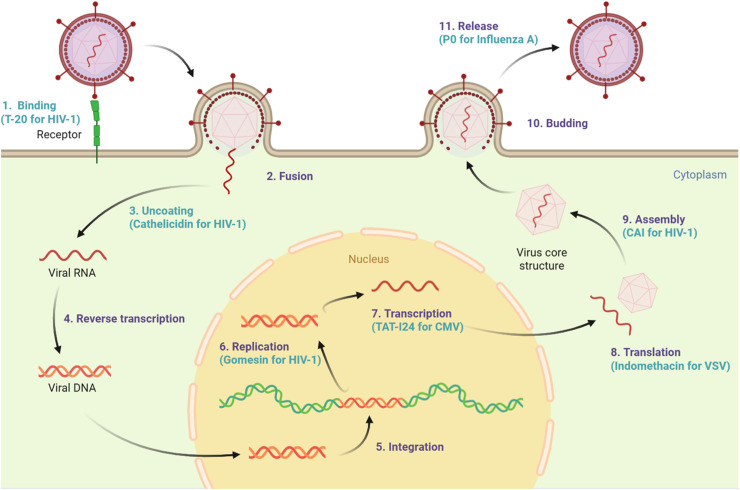
The effect of AVPs on different stages of the viral replication cycle.

This restructured framework clarifies how general antiviral strategies intersect with viral structural features, while integrating examples of AVPs that act at each stage of the viral life cycle (Agarwal and Gabrani, 2021; Qureshi et al., 2014) (Table 1 and Fig. 1).

**Table 1:** Mechanism of action AVPs.

No.	Mechanism of action	Enveloped Viruses (Examples)	Non-Enveloped Viruses (Examples)	Representative AVPs
1	Membrane/capsid disruption	Disruption of viral envelopes (*HIV, HSV, Influenza*)	Capsid destabilization (*Adenovirus, HPV*)	LL-37, melittin, cecropins
2	Inhibition of receptor binding & entry	Blockade of glycoprotein receptor interactions (*HIV* gp41, *Influenza* HA, *SARS-CoV-2* S)	Binding to viral capsid proteins preventing receptor-mediated entry (*Adenovirus, HPV*)	T-20, DP178/sifuvirtide, Pep 19–2.5
3	Interference with genome replication & protein synthesis	Inhibition of viral polymerase/proteases (*HIV, HCV, SARS-CoV-2*)	Inhibition of viral proteases, and genome replication (*Enteroviruses, Rhinoviruses*)	Hepcidin, protegrin-1, LVLQTM peptide
4	Inhibition of assembly & release	Blocked of viral budding and assembly (*HIV, Influenza*)	Prevention of capsid formation or genome encapsidation	Retrocyclins, CAP37
5	Immunomodulation	Induction of cytokines, activation of NK/macrophages, suppression of cytokine storm (*Influenza, SARS-CoV-2*)	Immune enhancement against non-enveloped viruses (*HPV, Adenovirus*)	Defensins, cathelicidins (LL-37)

### 3.1 AVPs against various viruses classical mechanisms

This section highlights AVPs that act on a range of viruses, including both enveloped and non-enveloped types.

#### 3.1.1 Activity against enveloped viruses

Membrane-active AVPs represent one of the most direct and effective classes of antiviral agents, particularly against enveloped viruses. Their amphipathic structures enable insertion into lipid bilayers, disruption of viral envelopes, or blockade of envelope protein-mediated fusion with host cell membranes. The antiviral efficacy of these peptides relies on either physical disruption of the viral membrane or molecular interference with viral glycoprotein host receptor interactions.

One of the most clinically advanced examples is T-20, a 36-residue peptide derived from the HIV-1 gp41 heptad repeat region, which prevents the formation of the six-helix bundle required for viral–cell membrane fusion (Kilby et al., 1998; Lalezari et al., 2003). Similarly, the EK1 peptide, designed from the HR1 domain of coronaviruses, demonstrates broad-spectrum fusion inhibition by binding to the HR1 region of spike proteins, thereby blocking viral entry in SARS-CoV, MERS-CoV, and SARS-CoV-2 (Xia et al., 2019).

Other naturally occurring membrane-active peptides include Melittin, from bee venom, and the human cathelicidin LL-37. Both display amphipathic α-helical structures that enable them to insert into viral envelopes, causing membrane destabilization and lysis in viruses such as influenza and herpes simplex virus (Tripathi et al., 2014; Lee and Lee, 2015). In addition, peptides mimicking host receptor motifs have shown promise. For example, ACE2-derived peptides can competitively block the binding of SARS-CoV-2 spike glycoprotein to the ACE2 receptor, preventing viral entry. Molecular docking and *in vitro* assays have validated their inhibitory activity, highlighting the therapeutic potential of host-receptor mimetics (Zhang et al., 2020).

Defensins are a well-characterized family of cationic host defense peptides with potent antiviral activity. Among them, human α-defensins (notably HNP-1, HNP-2, and HNP-3) have been shown to block HIV infection by binding to gp120 and CD4, thereby interfering with viral entry, and to inhibit replication of influenza and herpesviruses through interactions with viral envelopes and disruption of membrane integrity (Chang et al., 2005; Hazrati et al., 2006).

Similarly, θ-defensins (retrocyclins), cyclic peptides originally identified as truncated pseudogenes in humans but synthesized experimentally, exhibit broad antiviral activity. Retrocyclins prevent HIV-1 entry by blocking gp41-mediated fusion and can also aggregate viral particles, reducing infectivity (Cole et al., 2002; Wang et al., 2004).

In addition to entry inhibition, defensins have been reported to interfere with intracellular replication steps by targeting viral RNA or polymerase activity, providing a dual mechanism of antiviral action (Quiñones-Mateu et al., 2003; Klotman and Chang, 2006).

Collectively, these examples demonstrate that defensin-derived peptides represent a versatile class of AVPs capable of inhibiting both viral entry and replication across multiple enveloped viruses.

Together, these examples underscore the diverse strategies by which membrane-active AVPs interfere with the viral entry process. By either disrupting viral envelopes or competitively blocking fusion and receptor engagement, these peptides provide a powerful foundation for the development of broad-spectrum antivirals.

#### 3.1.2 Activity against non-enveloped viruses

Cationic peptides, which carry a positive charge from amino acids like arginine and lysine, have a strong antiviral effect on non-enveloped viruses (De Angelis et al., 2021). This charge allows them to attach to the negatively charged viral capsid, blocking viruses from entering host cells (Mulder et al., 2013).

On the other hand, non-cationic peptides are less effective against viruses because they carry a negative charge. Certain non-cationic AVPs from various sources have demonstrated their ability to combat non-enveloped viruses.

Both Cecropin B and the synthetic variant CF17 disrupt the capsids of non-enveloped viruses (Nicol et al., 2012). CAP37 also damages the capsid of *Adenovirus* (Eisenberg et al., 1985), while HD5 (Human alpha defensin) and RTD-1 prevent the binding and infection of *BK polyomavirus (BKV)* and *Papillomaviruses* by capturing and aggregating virions instead of disrupting the capsid (Serna et al., 2021; Wang et al., 2009).

The peptide Epinecidin-1 (Epi-1) counteracts foot-and-mouth disease virus (FMDV) by inhibiting the transcription of its viral genes in host cells (Duffalo and James, 2003).

A peptide sourced from bovine lactoferrin (bLfcin) stops *Adenovirus* from binding to HEp-2 cells and entering them (Liu et al., 2012).

LVLQTM, a short peptide with six amino acids, acts as a pseudosubstrate that interacts with the enzyme's substrate binding site, inhibiting human rhinoviruses (HRV) and human *Enterovirus* 71 (EV71) by blocking their viral cysteine protease 2A (2Apro) (Armitage et al., 2014; Krepstakies et al., 2012).

### 3.2 Modifications of AVPs to enhance activity

Chemical and structural modifications are widely applied to improve the potency, stability, and pharmacological properties of AVPs. These strategies include sequence truncation, residue substitution, cyclization, and conjugation with lipophilic or stabilizing moieties. The sequences of the important AVPs that have been studied the most and have shown promising results are presented in Table 2.

1. Peptide length reduction: For peptide length reduction, for example, the CHR-derived peptide SC29EK (29 aa) retains strong anti-HIV-1 activity comparable to its parental SC34EK (34 aa), and exhibits potent inhibitory capacity even against T-20-resistant HIV-1 strains, with EC₅₀s in the low nanomolar (nM) range. In contrast, further truncated versions (like, SC22EK, 22 aa) show substantially reduced activity (no significant inhibition up to ∼10 µM). These data indicate that reduction of 5–12 aa can be tolerated without large loss of potency only if critical structural motifs (like, EK repeats, pocket-binding residues) are preserved (Naito et al., 2009). This demonstrates that rational truncation can enhance membrane penetration while maintaining potency.
2. Lipid conjugation for stability: Modification with lipid moieties increases peptide–membrane interactions and serum half-life. A prominent example is EK1C4, a cholesterol-conjugated derivative of EK1 (36 residues). EK1C4 displayed a ∼149-fold increase in inhibitory activity against SARS-CoV-2 (IC₅₀ = 1.3 nM *vs.* 193 nM for EK1) due to improved membrane anchoring and stability (Xia et al., 2019; Xia et al., 2020).
3. Backbone and residue substitution. Incorporation of non-natural amino acids can enhance resistance to proteolytic degradation. For example, retrocyclins containing d-amino acid substitutions showed improved protease resistance while maintaining activity against influenza and HSV (Skalickova et al., 2015). Similarly, substitution of lysine with arginine in certain AVPs improved charge distribution and antiviral efficacy (Jenssen et al., 2006).
4. Cyclization. Head-to-tail or disulfide cyclization stabilizes peptide secondary structure, reducing susceptibility to proteolysis. Cyclic derivatives of θ-defensins and lactoferricin analogs have shown extended serum stability and enhanced antiviral profiles (Tamamura et al., 1999)

**Table 2:** Representative AVP sequences included in this review.

No.	AVP	Target virus	Sequence	Reference
1	LL-37	INFV	LLGDFFRKSKEKIGKEFKRIVQRIKDFLRNLVPRTES	(Tripathi et al., 2013)
2	T-20	HIV	YTSLIHSLIEESQNQQEKNEQELLELDKWASLWNWF	(Hu et al., 2019)
3	Cathelicidin	HIV	LLGDLLRKSKEKIGKEFKRIVQRIKDFLRNLVPRTES	(Wang et al., 2008)
4	Melittin	HIV	GIGAVLKVLTTGLPALISWIKRKRQQ	(Wachinger et al., 1998)
5	Cecropin	HIV	KWKLFKKIEKVGQNIRDGIIKAGPAVAVVGQATQIAK	(Wang, 2012)
6	Indolicidin	HIV, HSV	ILPWKWPWWPWRR	(Farr Zuend et al., 2018)
7	Retrocyclin-2	HIV, HSV	Cyclo(RRICRCICGRGICRCICG)	(Daly et al., 2007; Galdiero et al., 2013)
8	Theta-defensin	HSV	GICRCICGRGICRCICGR	(Yasin et al., 2004)
9	Dermaseptin	HSV	ALWMTLLKKVLKAAAKALNAVLVGANA	(Galdiero et al., 2013)

The peptides shown were selected based on the following criteria: (i) repeated experimental validation of antiviral activity in peer-reviewed studies, (ii) representative structural diversity (α-helical, β-sheet, cyclic, and hybrid classes), (iii) coverage of both enveloped and non-enveloped viruses, and (iv) availability of reproducible primary sequence data. These examples are not exhaustive but highlight well-characterized AVPs that serve as prototypes for further design and modification.

Overall, these examples illustrate that shortening peptide length does not universally enhance activity, and that stability can be significantly improved through conjugation, substitution, or cyclization. Thus, the design of AVP modifications requires a context-dependent balance between potency, stability, and bioavailability.

### 3.3 Computational and AI-Driven approaches in AVP discover

The development of AVPs has been greatly accelerated by computational strategies, evolving from conventional bioinformatics to advanced AI–driven pipelines. Classical approaches, such as molecular docking and molecular dynamics simulations, remain valuable for elucidating peptide protein interactions and validating binding stability. However, these methods are often limited by their reliance on predefined structural information and relatively high computational costs. Recent advances in AI and machine learning (ML) have transformed AVP discovery into a predictive and generative science. Tools such as AVP-IC50Pred and AVPdb employ ML algorithms to predict peptide activity based on physicochemical descriptors and sequence patterns, enabling rapid screening of large peptide libraries (Qureshi et al., 2014; Thakur et al., 2012). Beyond supervised learning, deep neural networks have been trained on high-dimensional sequence data to improve the accuracy of AVP prediction and classification (Charoenkwan et al., 2021). Recurrent Neural Networks (RNNs) and Long Short-Term Memory (LSTM) networks are particularly suited for peptide sequence analysis, as they capture long-range dependencies in amino acid chains, enabling improved prediction of antiviral activity and peptide stability (Hochreiter and Schmidhuber, 1997). These models have been successfully applied in modeling peptide–protein binding motifs and in generating sequence–activity relationships. Transformers and attention-based models, inspired by natural language processing (NLP), represent a breakthrough in biological sequence modeling. Pre-trained transformer architectures (like, BERT, ProtBERT, ESM) have been applied to peptides for tasks such as peptide–MHC binding prediction, peptide generation, and peptide–virus interaction mapping. Their ability to learn contextualized representations makes them superior to CNNs and RNNs for large-scale peptide datasets (Rao et al., 2019; Elnaggar et al., 2021). Ensemble learning methods, including random forests, gradient boosting, and stacked ensemble approaches, remain powerful for AVP classification when integrating heterogeneous features such as sequence descriptors, physicochemical indices, and structural features. These methods often outperform single algorithms by reducing overfitting and improving generalizability across viral families (Mach, 2001). Reinforcement learning (RL) has recently been adopted in *de novo* peptide design, where peptide candidates are iteratively optimized for multiple properties (binding affinity, stability, immunogenicity, toxicity). By defining reward functions based on biological constraints, RL enables adaptive exploration of peptide chemical space, accelerating the generation of clinically viable AVPs (Das et al., 2021; Popova et al., 2018). Together, these algorithms represent a comprehensive AI toolkit for antiviral peptide discovery, moving the field from simple predictive models toward multi-objective, generative, and translational pipelines. This expanded methodological landscape justifies the emphasis on “AI-Driven Design” in the manuscript’s title and underscores the novelty of current research directions (Table 3, Table 4).

**Table 3:** Selected case studies of AI applications in antiviral peptide (AVP) discovery and optimization.

No.	AI/ML Approach	Application	Case Study/Tool	Key Outcome
1	Machine Learning Classifiers	Screening peptide libraries for antiviral activity	AVP-IC50Pred, AVPdb	Enabled rapid identification of candidate AVPs against *HIV* and *Influenza*
2	Deep Neural Networks (transfer learning)	Prediction of antiviral activity in novel peptides	ProtBERT, TAPE	Improved accuracy in classifying *SARS-CoV-2* inhibitory peptides
3	RNN/LSTM	Sequence-based AVP prediction	Applied in peptide-protein binding motif modeling	Captured long-range dependencies in peptide sequences
4	Generative Adversarial Networks (GANs)	*De novo* peptide design	GANs integrated with MD simulations	Generated novel broad-spectrum antimicrobial peptides with antiviral potential
5	RL	Multi objective peptide optimization	RL-based frameworks for peptide design	Optimized peptides for antiviral potency and reduced toxicity
6	Ensemble Learning (Random Forest, Gradient Boosting)	Robust AVP classification	Applied in hybrid feature-based models	Reduced overfitting, improved generalizability across viral families

**Table 4:** Updated overview of major antiviral peptides, their target viruses, and mechanisms of action.

No.	Peptide	Target viruses	Primary mechanisms	Reference
1	Temporin L/ Temporins (amphibian)	*HSV-1 & HIV* (reported previously), *Influenza A (H1N1/H3N3), SARS-CoV-2* (*in vitro*), *Paramyxoviruses*	Amphipathic α-helical peptides membrane insertion → envelope disruption; inhibition of fusion	(Zannella et al., 2022; De Angelis et al., 2021)
2	LL-37 (human cathelicidin)	*Influenza, HSV, RSV, SARS-CoV-2* (*in vitro*)	Membrane disruption; immunomodulation (cytokine induction, innate activation)	(Tripathi et al., 2013)
3	EK1/EK1C4 (HR-derived, coronavirus fusion inhibitors)	*SARS-CoV, MERS-CoV, SARS-CoV-2* (broad activity)	HR1/HR2 interference → fusion inhibition; lipidation (EK1C4) increases membrane anchoring and potency	(Xia et al., 2019; Xia et al., 2020)
4	Enfuvirtide (T-20)	*HIV-1* (clinical)	Gp41-drived fusion inhibitor blocks six-helix bundle formation → prevents fusion	(Kilby et al., 1998; Lazzarin et al., 2003)
5	Retrocyclins/ Ɵ-defensins	*HIV, HSV, Influenza* (expanded evidence)	Capsid/viral surface binding, aggregation; some membrane effects; improved stability in cycle forms	(Cole et al., 2002; Daly et al., 2007)
6	Plectasin	*Dengue* (reported); broad antiviral potential reported *in vitro*	Interaction with viral proteins/ entry steps; also antibacterial origin	(Rothan et al., 2012)
7	Protegrin-1	*Dengue*, other RNA viruses (*in vitro*)	Protease inhibition; membrane disruption in some contexts	(Rothan et al., 2012)
8	Cecropins/ Melittin	*HIV, HSV, Influenza* (membrane active)	Strong membrane lytic activity → viral envelope disruption; fusion inhibition at sublytic concentrations	(Jenssen et al., 2006; Wachinger et al., 1998)

Beyond classical predictive pipelines, advanced generative AI approaches have recently emerged as powerful tools for AVP discovery. GANs have been applied to generate novel bioactive peptide sequences by learning latent representations of amino acid distributions and optimizing functional motifs (Popova et al., 2018). Similarly, LLMs trained on massive protein and peptide corpora, such as ProtBERT, ESM, and ProteinGPT, are increasingly used for *de novo* sequence generation, peptide–virus interaction prediction, and context-aware optimization of physicochemical properties (7, 8, 10). Furthermore, generative molecular design frameworks that integrate RL with multi-objective optimization enable adaptive exploration of peptide space while simultaneously accounting for stability, immunogenicity, and toxicity constraints (Das et al., 2021; De Bacco et al., 2018). These advances complement predictive models by not only evaluating existing peptides but also proactively generating new AVPs with tailored therapeutic potential. Such frameworks represent a paradigm shift toward adaptive, scalable, and XAI pipelines for next-generation peptide therapeutics. Our manuscript provides a future-oriented perspective that is largely absent from prior AVP reviews, which have focused mainly on classical bioinformatics or descriptive peptide characterization.

In addition to predictive tools, several AI-based platforms have been developed specifically for AVP discovery. AVPdb serves as a curated repository of experimentally validated peptides (Qureshi et al., 2014), while AVPpred and AVP-IC50Pred employ support vector machines and ensemble learning to predict antiviral activity in terms of IC50 values (Qureshi et al., 2014; Thakur et al., 2012). More recently, deep learning architectures have gained traction: LSTM networks capture long-range sequence dependencies for peptide–virus interaction modeling (Hochreiter and Schmidhuber, 1997), and Transformer-based language models such as ProtBERT and ESM achieve state-of-the-art performance in peptide classification and *de novo* sequence generation (Rao et al., 2019; Elnaggar et al., 2021). These platforms exemplify how AI is transforming peptide discovery from heuristic screening into predictive and generative science.

### 3.4 Interpretability and XAI in AVP discovery

A major limitation of applying AI to biomedical sciences, including AVP discovery, is the so called “black box” problem. While deep learning models such as CNNs, RNNs, and Transformers can achieve high predictive accuracy, the underlying decision-making process often remains opaque. This lack of transparency reduces scientific trust and poses barriers for regulatory approval and clinical translation. To overcome this challenge, several XA) approaches have been proposed:

Model-agnostic explanation tools such as SHAP (SHapley Additive exPlanations) and LIME (Local Interpretable Model-agnostic Explanations) can identify key amino acid residues, motifs, or physicochemical features that contribute most to a model’s prediction (Lundberg and Lee, 2017). Attention mechanisms in Transformers inherently provide interpretability by assigning weights to specific sequence positions, thereby allowing researchers to visualize which regions of a peptide drive predicted antiviral activity (Vig et al., 2020). Hybrid AI-mechanistic frameworks integrate ML models with traditional docking or molecular dynamics simulations, enabling both high-throughput prediction and biologically interpretable validation (Das et al., 2021). Visualization tools such as saliency maps and gradient-based methods are increasingly used to highlight important input regions, further bridging the gap between prediction and biological explanation (Simonyan et al., 2013).

Addressing the black box problem is particularly critical for AVPs, where understanding why a peptide is predicted to have antiviral activity can guide rational design, improve reproducibility, and build confidence among clinicians and regulators. Future work should therefore prioritize interpretable AI pipelines, ensuring that AI-driven AVP discovery is not only accurate but also explainable and trustworthy for translational applications. By explicitly highlighting interpretability, our review contributes a novel dimension to the field, positioning AI not only as a discovery engine but also as a transparent and translationally robust framework for antiviral peptide development.

## 4 Therapeutic potential, clinical trials and translational advances

The ability and promise of both natural and synthetic AVPs in combatting viral infections have generated significant interest. Due to their safety and minimal side effects, AVPs appear to be potential treatments for viral illnesses. These peptides can be administered in various ways, including orally, intravenously, via inhalation, or through topical ointments. In one study, LL-37 was nebulized and given to mice infected with influenza, leading to a reduction in disease severity (Gordon et al., 2009). Another investigation focused on lactoferrin and EDN for respiratory viral conditions like rhinovirus and *Respiratory syncytial virus (RSV),* yielding encouraging results in lessening disease severity (Gulati et al., 2019). For viral infections targeting internal organs, intravenous or oral delivery of AVPs is generally preferred. A research study looked into the effects of oral lactoferrin on HCV treatment, revealing that it was quite effective against the viral infection (Skeate et al., 2020). Additionally, AVPs can come as gels, creams, and ointments; for instance, human α-melanocyte-stimulating hormone (α-MSH) is currently undergoing clinical trials for its effectiveness in treating vaginal candidiasis (Huang et al., 2018). Furthermore, α-MSH also demonstrated antiviral properties against HIV (Di Biase et al., 2003), making it a potential treatment for sexually transmitted diseases like HSV. The use of AVPs in gel form may offer an affordable and efficient approach to address viral infections.

In a phase II trial, researchers explored the best dosage of PAC-113 for treating oral candidiasis in individuals with HIV (N. Falah et al., 2012). Meanwhile, a phase III trial is examining sifuvirtide to promote antiviral immune responses in HIV-infected patients (N. Falah et al., 2012). Initial findings from clinical trials on AVPs indicate they may serve as a useful complement in fighting viral infections. Nevertheless, additional research and clinical trials are required to effectively treat HIV using AVPs (Wang et al., 2008).

Moreover, AMPs have been extensively studied and tested against influenza viruses. An animal study involving mice examined a 27-amino acid peptide from the N-terminal region of human bactericidal/permeability-enhancing protein (BPI). This protein disrupted the viral envelope, thereby inhibiting *Influenza A virus* infections from strains H1N1, H3N2, and H5N1 (Wachinger et al., 1998). The most effective peptide identified for its antiviral action is Temporin L (TL), which has shown notable inhibitory effects against influenza virus, *Severe acute respiratory syndrome coronavirus 2* (SARS-CoV-2), herpesviruses, and paramyxoviruses. It also boasts low toxicity and demonstrated enhanced antiviral effects when lipidated (Daly et al., 2007). AMPs have yielded promising results in animal studies related to influenza, such as a lactoferrin study in mice infected with H5N1, which illustrated that lactoferrin possesses anti-inflammatory qualities and lessens intestinal harm (Galdiero et al., 2013). Despite these findings, further studies and clinical trials remain essential to fully explore the antiviral potential of AMPs against influenza infections. Clinical trials involving AMPs are detailed in Table 5.

**Table 5:** Clinical trials of AVPs.

No.	NCT Number	Status	Phase	Conditions	AVP
1	NCT00001118	Complete	I	*HIV* Infections	T-20
2	NCT00002363	Complete	I	*HIV* Infections	SCP3
3	NCT00002228	Complete	II	*HIV* Infections	T-20
4	NCT00856154	Complete	I	*HIV* Infections	Peptides on autologous DCs
5	NCT04560569	Unknown	II	*HIV* Infections	Albuvirtide
6	NCT00031044	Complete	I/II	*HIV* Infections	T-20/Amdoxovir
7	NCT04819347	Unknown	II	*HIV* Infections	Albuvirtide/3BNC117
8	NCT06799650	Recruiting	II	*HIV* Seropositivity	GammoraÂ®
9	NCT04421534	Unknown	II/III	*SARS-Cov-2*	Lactoferrin
10	NCT04375124	Complete	I	*SARS-Cov-2*	Angiotensin peptide (Agarwal and Gabrani, 2021; Wang et al., 2016; Jenssen et al., 2006; Zannella et al., 2022; Kilby et al., 1998; N. Falah et al., 2012; Rao et al., 2019)
11	NCT04771013	Complete	II	*SARS-Cov-2*	Thymic peptide
12	NCT04627233	Unknown	I	*SARS-Cov-2*	HEP-1
13	NCT04316377	Unknown	IV	*SARS-Cov-2*	Hydroxychloroquine Sulfate
14	NCT04330690	Unknown	III	*SARS-Cov-2*	Artesunate/Imatinib/ Infliximab/ Dexamethasone/LSALT Peptide
15	NCT04402957	Complete	II	*SARS-Cov-2, RSV*	LSALT peptide
16	NCT03354728	Recruiting	I/II	*CMV* Infection	Multi-peptide CMV-Modified Vaccinia Ankara Vaccine
17	NCT05841095	Recruiting	I/II	Chronic *Hepatitis B*	ISA104
18	NCT00602784	Complete	II	*HCV*	IC41
19	NCT04935801	Complete	I	*DENV*	NaNO-DENGU

### 4.1 Emerging topics and novel trends

In recent years, the landscape of viral infections has dramatically shifted to the emergence of novel pathogens and the resurrection of highly mutant viral strains. This trend has underlined the importance of comprehensive-spectrum and multi-target AVPs that can simultaneously prevent viral replication and modify host immune reactions (Brice and Diamond, 2020).

#### 4.1.1 AVPs against emerging viral threats

**SARS-CoV-2:** During the COVID-19 pandemic, several AVPs demonstrated promising inhibitory effects against SARS-CoV-2. Peptides derived from ACE2 receptor mimetics and fusion inhibitors targeting the spike (S) protein have shown the ability to prevent viral entry. Moreover, defensins and cathelicidins were found to both inhibit viral replication and regulate the inflammatory response associated with cytokine storm, suggesting their dual antiviral and immunomodulatory roles (Essa et al., 2022).

**MPXV:** The recent re-emergence of MPXV has renewed interest in AVPs capable of targeting orthopoxviruses. Early studies suggest that cationic AVPs can interfere with the viral envelope proteins essential for host entry. Given the similarities with vaccinia virus, peptide-based inhibitors originally developed for poxviruses could be rapidly repurposed against MPXV, highlighting the flexibility of AVPs in addressing zoonotic threats (Miah et al., 2022).

**Influenza variants:** The continuous evolution of influenza A and B viruses poses challenges for vaccine and antiviral development. AVPs such as FluPep and temporins have been shown to disrupt HA-mediated fusion and inhibit multiple influenza subtypes, including drug-resistant strains. Importantly, lipidated AVPs have demonstrated enhanced stability and potency against seasonal and pandemic influenza variants, underscoring their translational potential (Agamennone et al., 2022).

#### 4.1.2 Multi-Target and immunomodulatory AVPs

A particularly promising research direction includes multi-target AVPs that not only function on viral proteins, but also modulate the host immune system. For example:

• LL −37 antiviral disrupts viral membranes by increasing cytokine production.
• Defenseine induces viral aggregation and at the same time stimulate adaptive immunity.
• Engineer synthetic AVP with dual motifs can target viral fusion proteins and Toll-like receptors (TLRs), which leads to a combination of direct antiviral activity with immune activation.

These dual-function AVPs can provide better medical benefits by controlling viral replication while preventing excessive inflammation, an important factor in diseases such as COVID-19 and severe influenza (Gupta and Roy, 2021).

#### 4.1.3 Perspective on necessity AVPs

Emphasis on emerging viral threats highlights the immediate need for adaptable AVPs with comprehensive spectrum efficacy. The combination of multi-target activity with immunomodulation represents the next generation paradigm in antiviral peptide therapy. By addressing both viral replication and host immune dysregulation, AVPs may develop in universal antiviral platforms capable of combating future pandemics (Yang and Ma, 2024).

## 5 Translational and clinical applications

The clinical translation of AVPs requires not only proof-of-concept efficacy but also the establishment of scalable manufacturing, optimized delivery systems, and regulatory pathways. Many AVPs have already moved forward in preclinical and clinical studies, validating their medical therapeutic promises (Vargason et al., 2021).

### 5.1 Clinical trials and development pipeline

The translational pipeline for AVPs can be envisioned in four interconnected stages. First, *in silico* discovery leverages peptide databases (for example, AVPdb) and AI-driven prediction platforms (for example, AVP-IC50Pred, AVPpred) to identify candidates with favorable activity profiles. Second, preclinical validation involves *in vitro* antiviral assays and *in vivo* efficacy studies in animal models. Third, formulation and delivery optimization includes incorporation into nanoparticles, liposomes, or hydrogels to enhance bioavailability, protease resistance, and tissue targeting. Finally, clinical evaluation and commercialization encompasses Phase I–III trials assessing safety, pharmacokinetics, efficacy, and regulatory approval. This “bench-to-bedside” pipeline highlights the interdisciplinary integration of computational biology, nanomedicine, and clinical sciences in AVP development (Agarwal and Gabrani, 2021; Vargason et al., 2021).

Multiple AVPs, such as T-20 for HIV and lactoferrin-based formulations for respiratory viruses, have reached Phase II/III clinical trials (Vargason et al., 2021; Zhou et al., 2015). The clinical development pipeline of AVPs can be envisioned in four stages:

1. **Discovery and *in silico* prediction:** Identification of candidate AVPs through bioinformatics databases (like, AVPdb) and AI/ML-driven screening (like, AVP-IC50Pred, deep learning models).
2. **Preclinical validation:** *In vitro* testing for antiviral activity, cytotoxicity, and immunomodulatory effects, followed by *in vivo* efficacy studies in animal models.
3. **Formulation and delivery optimization:** Incorporation of AVPs into advanced carriers such as nanoparticles, liposomes, or hydrogels to improve bioavailability and stability.
4. **Clinical evaluation and commercialization:** Phase I–III trials assessing safety, pharmacokinetics, efficacy, and regulatory approval for clinical use.

This bench-to-bedside pipeline map underscores the interdisciplinary nature of AVP translation, combining computational biology, nanomedicine, and clinical science (Agarwal and Gabrani, 2021; Mousavi Maleki et al., 2022).

Several AVPs have advanced into human trials, underscoring their translational potential. T-20, an HIV fusion inhibitor peptide, remains the most well-established, having reached global approval following Phase III trials (Duffalo and James, 2003). Sifuvirtide, another HIV entry inhibitor, demonstrated strong efficacy in clinical evaluation (Yu et al., 2018). Lactoferrin-based formulations have entered Phase II studies for respiratory viral infections such as influenza and *RSV* (Harmsen et al., 1995; Van der Strate et al., 2001). In addition, PAC-113, a histatin-derived peptide, underwent Phase II clinical trials for oral candidiasis in HIV-infected patients (N. Falah et al., 2012). These clinical examples illustrate the feasibility of advancing AVPs from preclinical discovery to therapeutic application and reinforce their relevance as next-generation antivirals (Table 4).

### 5.2 Novel delivery systems for AVPs

A major obstacle for AVP translation is their instability and rapid decline by proteases *in vivo*. To overcome this, advanced delivery platforms have been developed:

• Nanoparticle-based systems: Polymeric and lipid nanoparticles (LNPs) protect AVPs from enzymatic decline, increase systemic circulation, and enable targeted distribution to infected tissues. For example, Nanoparticle-in capsulated AVP has increased antiviral efficacy in respiratory viral models (Chakravarty and Vora, 2021).
• Hydrogel Formulation: Injectable or topical hydrogel provides continuous release of AVP, especially beneficial for mucosal applications such as genital HSV or HPV infection (Malmsten, 2011).
• Intranasal and inhalable Formulations: AVP direct distribution for the respiratory tract enhances local concentration on infection sites (for SARS-CoV-2 and influenza), bypasses systemic decline and reduces dosage requirements (Cipolla et al., 2025).

These delivery systems not only improve medical efficacy, but also extend the clinical appropriateness of AVP to topical, oral, intravenous and breathable routes.

### 5.3 Commercial and regulatory perspectives

While some AVPs have entered late phase tests, challenges are in increasing peptide synthesis, ensure cost-efficiency, and meet regulatory standards for safety and efficacy. Good Manufacturing practice (GMP)-Cultivant synthesis and stability-growing modifications (for example d-amino acids, peptide cycles, lipidation) are important for commercial success. In addition, regulatory agencies are rapidly supporters of peptide-based medical, given their low toxicity and favorable safety profiles (Perico-Franco and Rosas-Pérez, 2025; Murugan et al., 2021).

### 5.4 Outlook

By integrating AI-driven discovery, nanotechnology-based distribution, and strong clinical pipelines, AVPs are ready to infection in clinically viable antiviral drugs from experimental molecules (Szymczak et al., 2025; Murugan et al., 2021). Their adaptability makes them not only attractive to treat current viral infections, but also to the rapid response to future epidemic.

## 6 Comparative landscape of AVPs

To better illustrate the therapeutic capacity of AVP, we propose a comparative summary that highlights their goals viruses, clinical development stages, benefits and challenges (Table 6).

**Table 6:** Comparative observation of representative AVP against various viral infections.

No.	AVPs	Target viruses	Advantages	Challenges	Reference
1	LL-37	*Influenza, RSV, HSV*	Broad-spectrum; antiviral + immunomodulatory	Rapid degradation; low systemic stability	(Tripathi et al., 2013)
2	T-20	*HIV-1*	Potent entry inhibitor; first-in-class AVP drug	High cost; injectable only; resistance development	(Foy and Juethner, 2004)
3	Lactoferrin-derived peptides	*SARS-CoV-2, HCV, Influenza*	Safe; oral/topical; anti-inflammatory activity	Variable efficacy across strains	(Ammendolia et al., 2012)
4	Temporins	*Influenza variants, HSV*	Potent activity *vs* resistant influenza; synergistic	Short half-life; needs formulation stabilization	(Zannella et al., 2022)
5	Sifuvirtide	*HIV-1*	Improved stability *vs* T-20; lower resistance risk	Limited global availability; parenteral use	(Yu et al., 2018)
6	Pep19–2.5 (Aspidasept®)	*HIV-1, HPV*	Blocks viral entry + modulates host receptors	Needs advanced delivery for systemic use	(Van der Strate et al., 2001)
7	Nanoparticle-encapsulated AVPs	*SARS-CoV-2, Influenza*	Enhanced stability; targeted delivery; inhalable	Complex manufacturing; regulatory hurdles	(Liu et al., 2023)

## 7 Future directions and perspectives

The current treatments for some viral infections are not very effective, making it essential to explore new therapeutic methods. Peptides show great potential as treatments since they are generally safe and have low toxicity. Research on naturally sourced cationic peptides indicates that AMPs are a viable option for addressing infections and are commonly applied in medicine.

However, there are challenges with AVPs, such as limited ability to cross membranes, degradation by enzymes, and their quick removal from the body. Therefore, it is important to create delivery systems and formulations that can address these issues (Nelson and Rosowsky, 2002). To effectively target AVPs, it is crucial to optimize the peptide sequence for proper binding to the intended cells (Chang and Yang, 2013). Using appropriate delivery methods like nanoparticles, cell-penetrating peptides, and liposomes can protect AVPs from degradation and enhance their stability, allowing them to reach the target cells effectively (Dwyer et al., 2007). Combining AVPs with antiviral medications that have complementary effects and support the immune system can create a strong strategy against viral infections (Sadler et al., 2002).

### 7.1 Advanced future directions

**Fig. 2:**
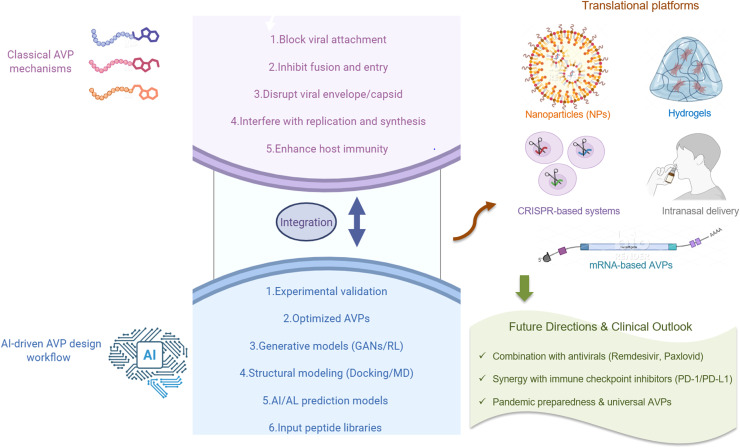
Next-Generation AVPs.

The next stage of AVP research extends beyond classical discovery and clinical testing towards integrated biotechnology platforms that can maximize therapeutic capacity. Many forward-looking strategies are estimated to redefine the role of AVP in infectious disease management (Navabhatra and Yangchaiya, 2025) (Fig. 2).

#### 7.1.1 CRISPR-Based delivery of AVPs

A transformative avenue includes CRISPR/Cas gene editing systems, which are capable of controlling engineer host cells and expressing AVPs in a continuous manner. By incorporating AVP-encoding genes in host genomes or epic vectors, CRISPR technique can create a long-term intracellular manifestation of medical peptides. Such an approach can provide continuous antiviral protection, especially for chronic viral infections such as HIV and HBV, where frequent viral reservoirs limit the effectiveness of traditional drugs. In addition, inducible CRISPR system AVP may allow temporary regulation of expression, which can reduce potential toxicity and improve medical precision (Getahun et al., 2022).

#### 7.1.2 mRNA-Based AVPs: lessons from vaccine platforms

Inspired by the success of mRNA vaccines against SARS-CoV-2, synthetic mRNA technology can be adapted to AVP delivery. In this strategy, the mRNA construction encoding optimized AVP is encountered in LNPs and distributed in host cells, where translation machinery produces active peptides. This approach combines the rapid adaptability of mRNA platforms with powerful antiviral properties of peptides, providing a scalable and modular solution against emerging viral outbreaks. Importantly, mRNA-based AVPs can enable on-demand peptide therapeutics, which are rapidly deployed in pandemic scenarios (Farshi, 2023).

#### 7.1.3 Synergy with existing antivirals and immunotherapies

AVPs rarely act in separation within biological systems. They can cause co -operative effects by mixing them with current antiviral drugs or immune checkpoint inhibitors (ICIs). For example:

• Combination with antivirals: AVPs can complement agents such as AVP Remdesivir and Paxlovid, which targets viral replication. This can reduce the emergence of multi-phase blockade resistant variants and increase medical efficacy (Jiang and Marraffini, 2015).
• Conjunction with ICIs: In chronic infections (for example HIV, HBV), where immune exhaustion is an obstacle, AVP co-administration with ICIs (like PD-1/PD-L1 or CTLA-4 inhibitor) can restore T-cell activity directly by stopping the T-cell activity (Fuchs et al., 2024).

#### 7.1.4 Toward universal antiviral platforms

By integrating CRISPR delivery, mRNA-based expression and combination therapies, AVPs may develop in universal antiviral platforms capable of addressing diverse pathogens. These strategies have the ability to remove the major boundaries of peptide therapeutics-such as volatility and short half life-as synthetic biology, nanomedicine and the strength of immunotherapy.

## 8 Conclusion

AVPs represent a versatile class of therapeutic candidates with the ability to target diverse viral families through multiple mechanisms, including membrane disruption, inhibition of receptor binding, blockade of genome replication, and immunomodulation. Compared with conventional small-molecule antivirals, AVPs offer several advantages such as broad-spectrum activity, low cytotoxicity, and reduced likelihood of inducing resistance. However, their clinical progress has been slowed by inherent limitations, particularly proteolytic instability, short half-life, and challenges in achieving efficient delivery to infection sites.

Our review highlights that advances in AI have fundamentally changed the landscape of AVP discovery. Predictive machine learning tools, GANs, LLMs, and reinforcement learning frameworks now enable de novo peptide design, optimization of stability and activity, and integration of multi-objective parameters such as toxicity and immunogenicity. Equally important, XAI approaches provide transparency, linking computational predictions to biological mechanisms and thereby enhancing translational trust.

Innovations in delivery systems including nanoparticles, hydrogels, intranasal sprays, and lipid-conjugated peptides have significantly improved AVP stability, bioavailability, and tissue targeting. Combined with promising results from clinical candidates such as T-20, sifuvirtide, lactoferrin formulations, and PAC-113, these advances demonstrate the realistic potential of AVPs to move from bench to bedside.

Critically, while the field has achieved major progress, most studies remain at the preclinical stage, and systematic evaluation in Phase II/III clinical trials is still limited. The integration of AI-driven discovery pipelines with novel delivery strategies represents a pivotal step to overcome these barriers. Looking ahead, synergistic approaches that combine AVPs with conventional antivirals or immunotherapies, as well as emerging modalities such as mRNA-encoded peptides and CRISPR-assisted delivery, may establish AVPs as frontline therapeutics against both existing and emerging viral threats.

In conclusion, AVPs are no longer merely experimental molecules but are increasingly becoming a strategic component of the antiviral therapeutic arsenal. By addressing current challenges through computational design and delivery innovations, AVPs have the potential to reshape the future of antiviral therapy and pandemic preparedness.

Data availability

All data generated or analysed during the study are included in the submitted manuscript information files. The raw data and data that support the findings of this study are available from the corresponding author upon request. There are no restrictions on data availability.

## CRediT authorship contribution statement

**Maryam Mashhadi Abolghasem Shirazi:** Writing – review & editing, Writing – original draft, Software, Resources, Project administration, Methodology, Investigation, Formal analysis, Data curation, Conceptualization. **Setareh Haghighat:** Writing – review & editing, Data curation. **Zahra Nikbakht:** Visualization, Software. **Elaheh Salimkia:** Visualization, Validation. **Armity Kiumarsy:** Visualization, Formal analysis.

## Declaration of competing interest

The authors declare that they have no known competing financial interests or personal relationships that could have appeared to influence the work reported in this paper.

## Data Availability

Data will be made available on request.
